# KONTAKT© for Australian adolescents on the autism spectrum: protocol of a randomized control trial

**DOI:** 10.1186/s13063-019-3721-9

**Published:** 2019-12-09

**Authors:** Bahareh Afsharnejad, Marita Falkmer, Melissa H. Black, Tasha Alach, Fabian Lenhard, Anna Fridell, Christina Coco, Kelly Milne, Nigel T. M. Chen, Sven Bölte, Sonya Girdler

**Affiliations:** 1 School of Occupational Therapy, Social Works and Speech pathology, Curtin University, Kent street, Bentley, Perth, WA 6102 Australia; 2 Curtin Autism Research Group (CARG), Curtin University, Perth, WA Australia; 3 CHILD, Swedish Institute for Disability Research, School of Education and Communication, Jönköping University, Gjuterigatan, Sweden; 4 Autism Association of Western Australia, Perth, WA Australia; 5 Center of Neurodevelopmental Disorders (KIND), Centre for Psychiatry Research, Division of Neuropsychiatry, Department of Women’s and Children’s Health, Karolinska Institutet & Child and Adolescent Psychiatry, Stockholm Health Care Services, Stockholm County Council, Stockholm, Sweden

**Keywords:** Social skills, Adolescents, KONTAKT, Autism spectrum disorder, Training

## Abstract

**Background:**

Individuals diagnosed with autism spectrum disorder (ASD) experience impairing challenges in social communication and interaction across multiple contexts. While social skills group training (SSGT) has shown moderate effects on various sociability outcomes in ASD, there is a need for (1) replication of effects in additional clinical and cultural contexts, (2) designs that employ active control groups, (3) calculation of health economic benefits, (4) identification of the optimal training duration, and (5) measurement of individual goals and quality of life outcomes.

**Method/design:**

With the aim of investigating the efficacy and cost-effectiveness of a SSGT, KONTAKT©, a two-armed randomized control trial with adolescents aged 12–17 years (*N* = 90) with ASD and an intelligence quotient (IQ) of over 70 will be undertaken. Following stratification for centre and gender, participants will be randomly assigned to either KONTAKT© or to an active control group, a group-based cooking programme. Participants will attend both programmes in groups of 6–8 adolescents, over 16 one-and-a-half-hour sessions. The primary outcome examined is adolescent self-rated achievement of personally meaningful social goals as assessed via the Goal Attainment Scaling during an interview with a blinded clinician. Secondary outcomes include adolescent self-reported interpersonal efficacy, quality of life, social anxiety, loneliness, face emotion recognition performance and associated gaze behaviour, and parent proxy reports of autistic traits, quality of life, social functioning, and emotion recognition and expression. Cost-effectiveness will be investigated in relation to direct and indirect societal and healthcare costs.

**Discussion:**

The primary outcomes of this study will be evidenced in the anticipated achievement of adolescents’ personally meaningful social goals following participation in KONTAKT© as compared to the active control group. This design will enable rigorous evaluation of the efficacy of KONTAKT©, exercising control over the possibly confounding effect of exposure to a social context of peers with a diagnosis of ASD.

**Trial registration:**

Australian New Zealand Clinical Trials Registry (ANZCTR). ACTRN12617001117303. Registered on 31 July 2017. anzctr.org.au

ClinicalTrials.gov, NCT03294668. Registered on 22 September 2017. https://clinicaltrials.gov

## Background

### Autism in adolescence

Autism spectrum disorder (ASD) is a condition of neurodevelopmental origin, presenting early in life [1]. Hallmark features of ASD include persisting challenges in social communication and interaction across multiple contexts, and restricted repetitive patterns of behaviour, interests, or activities, leading to qualitative impairment in significant areas of life [1, 2]. In 2015, it was estimated that approximately 0.6% of Australians had an ASD diagnosis, representing a 42.1% relative increase in prevalence from 2012 [3]. Prevalence rates vary across age groups, with the prevalence among Australian adolescents estimated to range from 3% to 4% [3]. The rising diagnostic trends for ASD among children over recent decades have seen an unprecedented number of adolescents with ASD, resulting in an urgent need for evidence-based interventions aimed at improving outcomes for these young people [4].

Adolescence is a time when social demands escalate as peer networks become increasingly important [5, 6]. Contrary to popular belief, most individuals with ASD might accurately perceive their level of social interaction and communication abilities and their limitations in networking with their peers [7]. The social difficulties associated with ASD during puberty [8], compounded by common comorbidities such as social anxiety, mean adolescence can be a particularly difficult period for these young people [9]. While impairments in daily functioning are central to a diagnosis of ASD [1], in adolescence it can have the complication of hampering the transition to adulthood, reducing adolescents’ desire for independent living and limiting education and employment outcomes [10, 11]. The cumulative impact of these experiences is evident in the poor quality of life outcomes observed during this period [12].

### Social skills group training

It has been argued that underpinning the core challenges of ASD is a paucity of social understanding [13, 14]. Social skills training, that is overtly teaching social skills, has been proposed as one means of ameliorating the difficulties of adolescents with ASD [15] with evidence showing a positive impact on social skills [16–18], anxiety [9, 19, 20], and family quality of life [21]. It is likely that participating in social skills training early in life will lessen and possibly even prevent social difficulties later in life [22].

While social skills training interventions can be delivered to individuals or groups, the group context has many apparent benefits for adolescents with ASD, including providing an in-vivo and immediate context for practising learnt skills and the opportunity for positive interactions with peers [7]. Social skills group training (SSGT) interventions for adolescents diagnosed with ASD are commonly delivered in small groups of 4–9 participants [16, 17, 20, 23–25] with intelligence quotients (IQs) of > 70, and led by one to three SSGT facilitators [15, 19]. Sessions aim to teach a range of skills promoting social communication and interaction skills, covering topics such as emotion recognition, assertion, initiation, interpretation of verbal and non-verbal cues, conflict management, coping strategies, self-control, cooperation, developing and maintaining a relationship, and strategies for handling bullying, rumours, and gossip [19, 24, 26].

A search of the current literature evaluating SSGTs for adolescents via randomized controlled trials (RCTs) identified a number of design and intervention limitations. Consistent design limitations included small sample sizes [9, 27–31], failing to employ blinded or external observer-report measurement of dependent variables [17, 20, 27, 29, 30, 32–35], failing to describe the randomization processes [27, 29–31, 33, 34] or employ allocation concealment [17, 20, 27–36], limited application of intent-to-treat data analysis [17, 27–30, 32–35], a lack of clarity in regard to compliance with the Consolidated Standards of Reporting Trials (CONSORT) statement [20, 27, 29, 30, 35, 36], and limited examples of mixed methods studies integrating quantitative and qualitative data collection methods and analysis in understanding the outcomes of interventions [17] (Additional file 1 and Additional file 2).

A range of identified intervention shortcomings included limited consideration or incorporation of adolescents’ personal goals [20, 27–31, 33, 34, 36], outcomes largely measured by parent proxy report [17, 28, 29, 31, 32, 35, 36], inadequate tailoring of interventions to individual activity preferences [20, 29, 30, 33–36], failing to consider the relationship between intervention dosage (that is the number of sessions) and outcomes [20, 27–31, 33–36], the absence of cost analysis [17, 20, 27–36], and limited reporting of adverse events during the intervention period [20, 27–30, 32–34, 36] (Additional file 1). While comparison groups varied considerably, most examined the efficacy of SSGT interventions relative to usual care waitlist control groups, with limited description of what interventions and therapies were received by comparison groups. To date, only three studies have compared the efficacy of SSGT interventions against an active control group [29, 30, 36], limiting understanding of the influence of confounding factors, such as exposure to social context, on the outcomes of SSGT interventions. As a result, it remains unknown whether the benefits of receiving a SSGT intervention result from significant exposure to a peer group or whether they can be attributed to the social skills training and psychoeducational components of these interventions.The perceived benefits of SSGT interventions from the perspective of the adolescents themselves in achieving their personally meaningful social goals has yet to be rigorously evaluated. While it is likely that researchers have been reluctant to focus on adolescent-determined personally meaningful outcomes, given the inherent difficulty adolescents with ASD experience in self-reporting their own and perceiving other’s emotional states [37, 38], there is a clear over reliance on parent proxy reports in research evaluating the efficacy of SSGT programmes [9, 17, 24, 35]. This is of particular concern given the level of discrepancy between adolescent self-report and parent proxy report ratings in the domains of emotional and social functioning [39]. The role of SSGT programmes in supporting the achievement of personally meaningful social goals of adolescents diagnosed with ASD remains unknown.

### Present study

While the KONTAKT© SSGT programme is designed for both children and adolescents, the present study will evaluate the efficacy of KONTAKT© in Australian adolescents. KONTAKT© originated in Germany [40–42], and was subsequently further developed and evaluated in Sweden via RCTs [17, 32]. In Sweden, the efficacy of KONTAKT© was examined in comparison to a treatment-as-usual control group receiving ongoing treatment only. Swedish adolescents receiving 12 sessions of KONTAKT© demonstrated improvements in social skills, particularly in girls, and improvements in adaptive behaviours as reported by parents immediately following the intervention [19]. Adolescents receiving the longer 24-session version of KONTAKT© demonstrated greater effects [32] than those receiving the 12 sessions, with effect sizes of 0.8 and 0.3 observed for the long and short versions, respectively, for the primary outcome measure [17].

As ASD is primarily associated with challenges in social skills functioning in a given context, it is critical that any social intervention considers the role of culture [43, 44]. To be truly effective, social skills training interventions must be tailored to reflect the cultural norms of a target group [45]. Given the cultural similarities between Sweden and Australia [46], it was deemed likely that KONTAKT© would be similarly effective in improving the social skills of Australian adolescents with ASD as it had been for their peers in Sweden. While the majority of the content of KONTAKT© was potentially directly transferable to the Australian context, in adapting it for the Australian context it was firstly translated from Swedish to English and modified for obvious cultural differences.

Given the findings from KONTAKT© evaluation studies in Sweden demonstrating that the 24-session version was significantly more effective for adolescents with ASD than the shorter 12 session version, a finding linked with the opportunity to participate in more individualized sessions and opportunities to practise skills [47], it was initially envisaged that the Australian evaluation would employ an adapted 24-session version. However, following consultation with Australian service providers and clinicians, it was determined that given the structure of the Australian school year, which is roughly standardised nationally to run across four terms of approximately 10 weeks each, a 16-session version of KONTAKT©, involving eight sessions per term across two consecutive school terms would be more feasible. The feasibility and acceptability of the 16-session version of KONTAKT© was assessed and demonstrated to be favourable in a pilot study (under review). This pilot study provided feasibility and preliminary evidence of the potential efficacy of the 16-session programme, supporting the achievement of the personal meaningful social goals of Australian adolescents with ASD (*p* < 0.001) immediately after the programme had ended. Based on the results from the pilot study, KONTAKT© was further refined in a collaboration between Swedish and Australian clinicians and researchers, and Australian adolescents with ASD and their parents. This process resulted in a standardised version of KONTAKT© for an Australian context in preparation for evaluating its efficacy in an RCT.

## Methods

### Aim

This protocol aims to employ an RCT design to rigorously evaluate the efficacy of KONTAKT© in supporting the attainment of personally meaningful social goals of adolescents with ASD. The design of this study will address many of the noted limitations of previous SSGT evaluation research, including implementation of a manualised intervention KONTAKT©, controlling for social context, employing a primary outcome measure with adequate power to assess the achievement of adolescents’ personally meaningful social goals, undertaking a cost utility analysis, and investigating the relationship between dose (number of sessions) and the response of adolescents with ASD to a SSGT intervention. The study design stipulates clear inclusion and exclusion criteria, standardised outcomes measures validated for use with adolescents with ASD, blinded assessment of outcome measures, and stratified randomization. This study seeks to answer two research questions (1): can KONTAKT© make a unique contribution to facilitate achievement of adolescents’ personally meaningful social goals above and beyond any support provided by the positive social context? (2) Is KONTAKT© cost-effective in comparison to an active control condition?

### Design

**Fig. 1:**
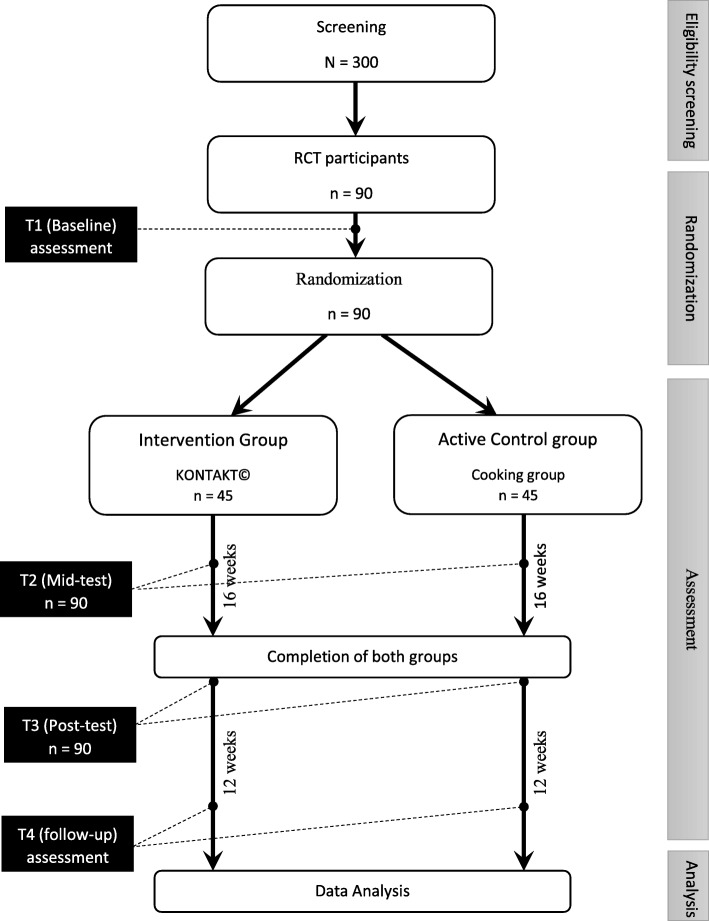
Participant recruitment, allocation, and assessment procedure

This will be a stratified (gender, site), RCT of a the SSGT programme (KONTAKT©) compared to a manualised active control group (a cooking group “Super Chef”) with a ratio of 1:1, adhering to the CONSORT statement for conducting high-quality RCTs [48] (Fig. 1). Data collection will occur at three time points (1): at baseline, prior to randomization to intervention or control; (2) at post-test, immediately following the intervention period; and (3) at follow-up, 12 weeks following the intervention (primary endpoint).

### Participants

Participants will be recruited through the Autism Association of Western Australia (AAWA) with the study promoted via newsletters, parent events, and social media. Informed consent/assent will be obtained from both parents and adolescents after receiving detailed verbal and written information about the programme, directions in case of adverse events, assessment timelines, and data collection procedures from a member of the research team at Curtin University.

### Inclusion criteria

The inclusion criteria for participating in this study will be as follows: (1) a clinical diagnosis of autism, Asperger syndrome, pervasive developmental disorder - not otherwise specified, or ASD according to the *Diagnostic and Statistical Manual of Mental disorders- version IV* (DSM-IV) [49], or ASD according to DSM-5 [1]. This will be further confirmed by administering the Autism Diagnostic Observation Schedule (ADOS-2) [50]; (2) an IQ > 70 on the Wechsler Abbreviated Scale for Intelligence (WASI-II) [51]; and (3) aged between 12 and 17 years at randomization.

### Exclusion criteria

Participants meeting the following criteria will be excluded from this study: (1) rule breaking and aggressive behaviours as confirmed by the Childhood Behaviour Checklist (CBCL) [52]; (2) prior clinically assessed, self-injurious behaviour; (3) low intrinsic motivation to participate; (4) insufficient English language skills; or (5) a history of clinically assessed self-injury, conduct disorder, hyperkinetic conduct disorder, antisocial personality disorder, borderline personality disorder, or any form of schizophrenia or related psychotic disorder that would interfere with participation or require alternative treatment.

Participants with common comorbid neurodevelopmental and psychiatric conditions such as attention problems, anxious or depressed behaviours as measured by CBCL [52] are acceptable in this trial as in previous evaluations of KONTAKT (c) [17, 32]. In addition, the participants may continue with their usual ongoing treatments or interventions.

### Sample size calculations

The KONTAKT© study in Sweden (both 12-week and 24-week versions) employed the Social Responsive Scale - second edition (SRS-2) as the primary outcome measure. Based on an effect size of 0.54, derived from roughly averaging the effect size (ES) as measured by the SRS-2 from trials examining the efficacy of the long 24-session (ES = 0.76) and the short 12-session (ES = 0.32) versions of KONTAKT© at post-test, and applying multivariate analysis of variance (MANOVA) for repeated measures (within-between interactions) at the three time points (using the intent-to-treat approach), a minimum of 57 participants are required (as calculated by G*Power [53] with power of 0.95 at a conventional error probability (α = 5%)). However, unlike the Swedish study, the present study will employ the Goal Attainment Scaling (GAS) as the primary outcome. It has been argued that GAS has good reliability when used as an outcome measure with interventions for adolescents with ASD [54–56]. Further, unlike previous investigation of KONTAKT© [17, 32], the current study will compare this SSGT efficacy against an active control group, to control for exposure to a social context with peers with ASD. It is likely that both these factors will have a limiting impact on the power of the study, and as such we will aim to recruit a sample of at least 90 participants into this study, increasing the likelihood of detecting possible effects [19]. This sample size will also account for an attrition rate of 37%, which is larger than what is expected based on the previous KONTAKT© studies.

### Setting

Participants expressing an interest in the study will be invited to a screening session and following determination of their eligibility will take part in a baseline assessment in a university laboratory at Curtin University, Perth, Western Australia. Both the KONTAKT© group and the active control cooking-group will be delivered by AAWA in one of their four metropolitan centres in Perth, Western Australia. The AAWA is the leading service provider for people with ASD in Western Australia, and the only specialist organisation providing a full range of services for children and adults in Australia, with over 700 multi-disciplinary staff.

### Randomization

Participants will be stratified for gender and then randomly allocated to either KONTAKT© (intervention group) or the Super Chef cooking-group (active control group) across AAWA centres. The randomization will be conducted by a statistician and sent directly to the AAWA study coordinator, supporting blind assessment of outcome measures by the research team.

### The interventions

In evaluating the feasibility and acceptability of KONTAKT© in an Australian context, a pilot version of the 16-session KONTAKT© was delivered to 16 adolescents meeting the inclusion criteria across 16 sessions in 20 weeks, with two 8-week session blocks interspersed with the Australian school holidays. After completing the KONTAKT© intervention, focus groups were held with participants, parents and trainers, capturing their perspectives of the programme. Following analysis of focus group data, final adjustments and modifications were made to the KONTAKT© 16-week variant, standardising the intervention in preparation for RCT evaluation. Tables 1 and 2 detail the structure and content of KONTAKT© (the opportunity to choose social themes/activities that reflect the participants’ personal goals or interests) and Super Chef (personal tastes for each recipe) sessions, and their emphasis on individualized activities aiming to promote motivation in the participants and generalization of skills [32].

**Table 1:** The structure, objectives and individualized parts of weekly KONTAKT© sessions [57]

Rounds	Objective	Individualized activity
Opening	Warm-up activity, initiating conversation, promoting interaction between group members, promoting eye contact	
Reviewing homework	Reinforcing and providing feedback, troubleshooting if necessary	Sessions 2–15
Group discussion	Exchanging experiences, promoting social cognition and social relationship	Sessions 12–15
Group activities	Providing practical solutions and strategies for everyday challenging social situations, fostering a feeling of group cohesion, practicing cooperation, practising recognising and interpreting non-verbal signals, eye contact, and facial expressions	Sessions 12–15
Snack time	Practising small-talk and turn-taking in unstructured conversations	
Assigning new homework	Generalizing learnt skills to everyday social situations	Fixed: sessions 1–10 Flexible: sessions 11–14
Closing	Evaluating the session, promoting interaction between group members

**Table 2:** The structure, objectives and individualized activities of weekly Super Chef sessions

Rounds	Objective	Individualized activity
Transition	Self-regulation and arrival into the session	
Activity 1	Sharing cooking experiences	
Activity 2	Preparation for cooking and food exploration	Every session
Snack time	Practicing small-talk and turn-taking in unstructured conversation, participating in games and activities	
Activity 3	Cooking or baking	Every session
Eating the prepared meal	Rating the prepared meal	
Clean up	Washing up, drying dishes, wiping down benches and tables, and sweeping the floor.	Every session
Transition	Recapping the session and feedback to the parents	

The Australian adaptation of KONTAKT©, employing a 16-session variant, aims to improve participants’ communication and social interaction skills, ASD-related traits, and the ability to empathise and adapt in a group setting of 6–8 adolescents aged 12–17 years [19, 21]. Groups meet weekly for an hour and a half, with two trainers delivering a programme underpinned by the principles of cognitive behaviour therapy, behaviour activation, observational learning, psychoeducation, and social cognition training [41, 42]. Sessions scaffold knowledge of common social rules and norms, aiming to promote problem-solving strategies, emotion recognition, and emotion expression [19].

KONTAKT© requires that at least one group trainer is a clinician with extensive experience working with children/adolescents with ASD, who has also received methodological training, or certification in KONTAKT© prior to the programme. Prior to the pilot, Australian clinicians from AAWA were trained by a Swedish team of certified KONTAKT© trainers. Requirements of KONTAKT© training certification include passing this method training, leading at least one KONTAKT© group under supervision, and achieving intervention fidelity as assessed by a KONTAKT© supervisor on the basis of submission of a filmed KONTAKT© session. A KONTAKT©-certified trainer can, in turn, instruct others in delivering KONTAKT©. In the present study, fidelity to the KONTAKT© intervention will be systematically assessed by trainers completing a session by session fidelity checklist, enabling an assessment of intervention fidelity. In addition, attendance sheets will be kept to record the participants’ compliance with the programme, with 80% attendance considered as compliant.

Super Chef is a manualised cooking-group programme specifically designed for this study (Table 2), with the goal of enabling comparison of KONTAKT© with an active social control group, enabling independent evaluation of the contribution of KONTAKT© to intervention outcomes. As in KONTAKT©, participants allocated to the Super Chef programme will meet weekly in groups of 6–8 for an hour and a half in a 16-session programme moderated by two trainers, one of which will be an occupational therapist with previous experience of working with Australian adolescents with ASD. As with KONTAKT©, each Super Chef session adheres to a specified agenda including discussions, taste testing, individual and group activities, snack time, cooking recipes, eating and rating recipes and cleaning up as rostered. Super Chef was developed by a team including occupational therapists with both clinical and research experience in working with adolescents with ASD, with consideration of the common sensory issues associated with ASD. Fidelity to the Super Chef intervention will be assessed via a fidelity checklist, specially designed for this programme, enabling assessment of the extent to which trainers followed the format of each session.

### Data collection

The data for this study will be collected by the measures as outlined in Table 3, at three time points, by an assessor blind to group allocation (1): before the intervention period (pre-intervention) (2); following the completion of the 16-week intervention (post-intervention); and (3), at 12 weeks following the intervention (follow up), with follow up being the primary endpoint. Additionally, there will be an 8-week data collection point for the cost analysis study (mid-intervention) (Table 3).

**Table 3:**
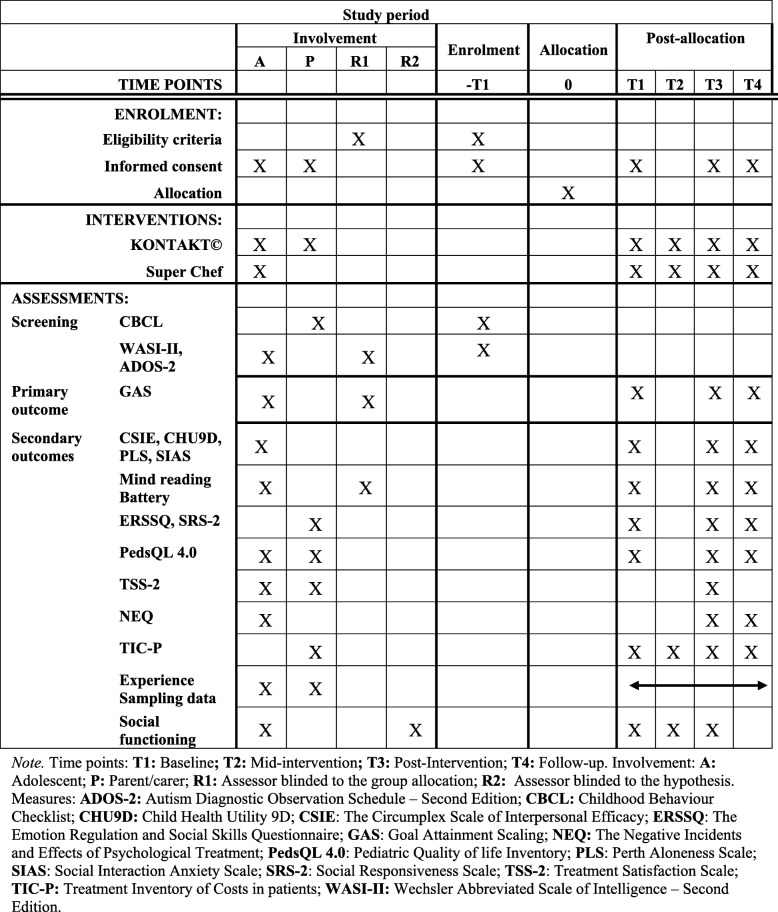
Schedule of enrolment, interventions, and assessments

Timepoints: *T1* baseline, *T2* mid-intervention, *T3* post-intervention, *T4* follow up. Involvement: *A* adolescent, *P* parent/carer, *R1* assessor blinded to the group allocation, *R2* assessor blinded to the hypothesis. Measures: *ADOS-2* Autism Diagnostic Observation Schedule - Second Edition, *CBCL* Childhood Behaviour Checklist; *CHU9D* Child Health Utility 9D, *CSIE* Circumplex Scale of Interpersonal Efficacy, *ERSSQ* Emotion Regulation and Social Skills Questionnaire, *GAS* Goal Attainment Scaling, *NEQ* Negative Incidents and Effects of Psychological Treatment, *PedsQL 4.0* Pediatric Quality of life Inventory, *PLS* Perth Aloneness Scale, *SIAS* Social Interaction Anxiety Scale, *SRS-2* Social Responsiveness Scale, *TSS-2* Treatment Satisfaction Scale, *TIC-P* Treatment Inventory of Costs in patients, *WASI-II* Wechsler Abbreviated Scale of Intelligence - Second Edition

Given this study will use an intent-to-treat approach, if participants are unable to finish the programme or attend the face-to-face assessment sessions, they will be contacted and encouraged to complete the questionnaires via email.

### Primary outcome measure

Goal Attainment Scaling (GAS) evaluates the outcomes and suitability of an intervention in an individual or group setting [58, 59]. Following the guidelines suggested by Kiresuk and colleagues (1994) adolescents randomized to both the treatment and control group, will establish at least three personally meaningful and measurable social goals in collaboration with an assessor blinded to the group allocation*.* The achievement of these goals will be scored via the GAS scoring system, whereby −2 indicates the participant’s current level of performance, −1 indicates less than expected, 0 indicates the expected progress, and +1 and +2 indicate progress above the expected level [60]. As suggested by previous research, the reliability of GAS will be calculated for this sample [54].

### Secondary outcome measures

The Social Responsiveness Scale - Second Edition (SRS-2) standard version is a 65-item parent rating scale, designed to measure autistic-like traits in individuals aged 4–18 years. The SRS-2 enables calculation of a total score and five subscales: social awareness, social cognition, social communication, social motivation, and restricted interests and repetitive behaviour. The scale is scored on a 4-point Likert scale, ranging from “not true” (0) to “almost always true” (3). Scores range from 1 to 195 with the expected value for individuals with a primary diagnosis of ASD being approximately 100 [61]. Previous studies in individuals with ASD show good psychometric properties for SRS-2 (internal consistency of 0.9) [62, 63]. As recommended for research, the raw scores of the measure (total and subscale) will be used in this study [63].

The Circumplex Scale of Interpersonal Efficacy (CSIE) [64] measures an individual’s confidence in their ability to successfully perform behaviours associated with each facet of the interpersonal circumplex (assert, distance, yield, and connect). Each octant scale shows a progressive blend of two axial dimensions (e.g. “speak up” representing an assertive action, “get them to leave me alone” a distancing action, and “tell them when I am annoyed” combining these two actions) [65]. As suggested by previous research, this study will use these dimensional scores instead of the eight octants to predict the outcome [66]. Previous studies in adolescents with ASD have demonstrated acceptable internal consistency of 0.78 for this measure [64].

The Perth Aloneness Scale (PLS) is a self-report measure consisting of 24 statements such as “I feel left out of things at school”, or “I get along with my classmates”, measuring four dimensions of loneliness in young people (isolation, friendship, and positive and negative attitudes toward solitude) [67–69]. Responses are recorded on a 6-point Likert scale indicating agreement with a statement, ranging from “never” (1) to “always” (6), with higher scores suggesting higher levels of loneliness and negative attitude towards solitude. This scale has yielded good reliability for the overall scale and subscales (Cronbach’s alpha = 0.84). The use of the scale in the current study is further supported by established norms for Western Australian adolescents [67].

The Emotion Regulation and Social Skills Questionnaire (ERSSQ) is a 27-item measure assessing emotion regulation and competency in social skills [70]. The questionnaire is designed to measure frequencies of effective engagement in social behaviours (e.g. “chooses appropriate solutions to social problems” or “deals effectively with bullying”), examining the competency of these skills [70]. Responses are rated on a 5-point Likert scale, ranging from “never” (0) to “always” (4), yielding a total score of 0–108, with higher scores indicating higher competencies in social behaviour. ERSSQ has demonstrated good internal consistency for children with ASD (Cronbach’s alpha = 0.89) [70].

The Paediatric Quality of life Inventory ™, version 4.0 (PedsQL ™ 4.0) is a 23-item parent proxy report and an adolescent self-report measure of adolescent’s quality of life underpinned by the four subscales of physical, emotional, social, and school functioning [39, 71]. Responders rate items according to if they have been a problem for them, on a 5-point Likert scale ranging from “never” (0) to “almost always” (5), with lower scores indicating better quality of life. Although there is no ASD-specific module available, the questionnaire has high validity and reliability (Cronbach’s alpha = 0.97) and has been used in adolescents with ASD [12, 39], including Australia youths with ASD [72, 73].

The Social Interaction Anxiety Scale (SIAS) is a 20-item measure assessing adolescents’ self-reported anxiety in social situations, via items such as “I become tense if I have to talk about myself” or “I find it easy to make friends my own age”. Items are rated on a 5-point scale ranging from “not at all” to “extremely”. Total scores range from 0 to 80 with higher scores indicating greater anxiety in social situations. The scale has a good internal consistency and test-retest reliability (Cronbach’s alpha = 0.94) [74] and has been validated in an Australian setting in Australian adults [75].

The Child Health Utility 9D (CHU9D) is a 9-dimension health-related quality of life scale (worried, sad, pain, tired, annoyed, school work, sleep, daily routines and activities), designed to estimate the adolescent’s quality-adjusted life years (QALYs), providing a standardised measure of disease burden. The measure is rated on a 5-point scale with a “don’t” sentence linked with no problems (e.g. “I don’t feel sad today”) and “very” with the participant experiencing many problems (e.g. “I feel very sad”). Calculation of a universal score is supported by an adolescent-specific scoring algorithm, with 1 representing “full health” and 0 “death” [76]. Previous research suggests that the CHU9D supports appropriate calculation of QALYs [77].

Healthcare consumption and productivity loss will be measured via a tailored version of the Trimbos/iMTA questionnaire for patients with a psychiatric disorder (TiC-P), a well-established questionnaire examining health care usage and any work, education, and productivity losses incurred by participants and their carers. The modified version of the TIC-P employed in this study comprises six sections enquiring about healthcare visits, support received both at and outside of school, medications and supplements, work, and education and productivity losses incurred by both parents and adolescents. The feasibly of the inventory was evaluated in the KONTAKT© pilot study.

The Mindreading Battery enables assessment of facial emotion recognition accuracy [78], with this study measuring adolescents performance across 40 basic and complex emotions, over 6 developmental levels with level 1 being the simplest (e.g. happy) and level 6 being the most complex (e.g. exonerated) (Table 3). Emotions are displayed in the form of 2–5-sec silent coloured video clips, with four multiple choice options, one of which is the correct emotion label and three of which are distractor items. The distracter options were randomly selected from the entire Mindreading Battery emotion groups, excluding the emotion group from which the target stimuli originated. Further details of the stimuli are outlined in Table 4. During the presentation of stimuli, eye-tracking data will be recorded via a remote eye tracker device (RED) developed by SensoMotoric Instruments, enabling examination of fixation patterns and fixation durations [79]. While the eye tracker accommodates small head movements, a chin rest will be available to participants who find it hard to sit still. Outcome measures will be assessed in relation to accuracy, response time, and number of and duration of fixations to dynamically defined areas of interest including the eyes, nose, and mouth of the stimuli [80, 81].

**Table 4:** Overview of the Mindreading Stimulus Battery

Stimuli characteristics	Number of stimuli items
Valence	
Negative	22
Positive	16
Gender	
Male (Pre)	17
Male (Post)	18
Female (Pre)	21
Female (Post)	20
Emotion level	
Basic	6
Level 1 and 2	6
Level 3 and 4	15
Level 5 and 6	11

The Negative Incidents and Effects of Psychological Treatment (NEQ) assesses potential adverse and unwanted events associated with attending the groups at the completion of each programme, via adolescent self-report [82]. The NEQ is a 32-item questionnaire requiring adolescents to quantify, on 5-point Likert scale with response options ranging from “not at all” to “extremely”, any negative events experienced during the intervention period, asking participants to attribute their causality to either the programme or external circumstances. Analysis of the measure has shown good reliability (Cronbach’s alpha = 0.95) [82].

The Experience Sampling Method (ESM) will evaluate adolescents’ everyday quality of life via daily responses from both adolescent and parent proxy report [83–85]. This 5-item measure, specifically designed for the purposes of this study, asks “In the last 24 hours, on a scale of 1 to 10 I have been feeling … ” with answers rated on a 10-point scale in five dichotomised emotional sets (sad/happy, lonely/unlonely, angry/calm, scared/unafraid, and anxious/confident). Questions are texted via mobile phones to adolescents and parents once daily from commencement of the groups to the final follow-up time point. The feasibility of this approach was assessed during a pilot study, with this method previously showing consistency across experiences and in examining the effect of social context on the daily experiences of adolescents with ASD [86].

The Treatment Satisfaction Scale (TSS-2) [87] is a short, 6-item parents’ and adolescents’ self-report instrument, measuring satisfaction with group attendance. Each item is scored on a 4-point Likert scale with response options ranging from “yes, very much” to “no”, with an open comment section, encouraging participants to freely share their experiences with the intervention.

Blind expert rating of social functioning will be assessed by an occupational therapist or psychologist, experienced in working with adolescents with ASD, and blinded to the study hypothesis, via a rating scale designed specifically for the purposes of this study. The scale requires a rating of participant’s social communication and interaction on a scale of 0–10 as observed during three video recordings of the snack times of sessions 2, 10, and 15 in both the intervention group and control groups presented in random order.

### Process evaluation

In determining the usability and the facilitators and barriers to both the KONTAKT© and Super Chef programmes, and the factors likely impacting their efficacy, a process evaluation will be undertaken. Parents, adolescence, and trainers’ feedback on both programmes will be sought via semi-structured interviews at the completion of the programmes. This will provide an in-depth understanding of those factors influencing the relative efficacy of both the KONTAKT© and Super Chef programmes.

### Statistical analyses

As suggested by previous research, reliability of the GAS goals will be investigated via examination of their measurability, equidistance, and difficulty [57]. A random effects regression model will be used to explore those factors associated with the raw data on the GAS scores (dependent variable), over the 9-month duration of the study. Independent variables for the model will be time, group (KONTAKT© versus Super Che’), age, IQ, gender, centre, and comorbidity as fixed effects, with follow up being the primary endpoint of the study. The random effect will be the participant’s ID number, thus accounting for the correlation between measurements made on the same individual within the model.

Analysis of secondary outcomes (interpersonal efficacy, quality of life, social anxiety, loneliness, facial emotion recognition, and eye-tracking behaviour) will be conducted in a similar manner (random effects regression model). Analysis will be based on an intent-to-treat approach [84], considering each participant as belonging to the study group they were initially allocated, regardless of treatment actually received. Missing data will be accounted for according to the guidelines specified for each measure; if no guidance is provided, missing data will be handled in accordance with the CONSORT statement for conducting high-quality RCTs [48]. Data analysis will be conducted using the SPSS version 24 statistical software [88].

The outcomes of the present study will be compared to results obtained by previous evaluations of the short and long variant of KONTAKT© undertaken in Sweden.

### Cost data analyses

Cost data will be analysed from both societal and healthcare perspectives, including the direct costs of the two experimental interventions, healthcare costs, and societal resource costs. Individual participant costs will be estimated and accumulated over the period of the study (9 months including follow up). Non-normality of the cost data can be assumed, and therefore missing data will be analysed using a non-parametric imputation model based on random forest estimation. CHU9D scores will be converted to QALYs using previously validated algorithms [76, 89]. The cost differences between the two experimental groups over all time points will be analysed by linear regression, using non-parametric bootstrapping with 5000 repetitions for the estimation of adequate confidence intervals, accounting for the skewedness of the data. Cost differences between the two groups will be presented in Australian dollars. As a global measure of cost-effectiveness and in line with international standards, the incremental cost-effectiveness ratio (ICER) will be presented [89], representing the additional cost of one additional QALY when participants receive KONTAKT© instead of Super Chef. The same analysis will be conducted for the ICER for treatment response that is the additional cost for one responder. To assure robustness of the results, sensitivity analysis will be conducted by increasing the costs of KONTAKT© by 25%.

## Discussion

The present paper describes the design of the KONTAKT© study, a RCT evaluating the efficacy of a 16-week SSGT intervention for adolescents with a confirmed diagnosis of ASD compared to Super Chef, a manualised active control group. KONTAKT© is an intervention that is theoretically based [21] and draws on previous research conducted with both German [41] and Swedish adolescents [32] with ASD. The design of the proposed study is robust, given it addresses many of the design limitations noted in previous research.

### Outcome measures

To date, the majority of studies evaluating the efficacy of SSGT interventions in adolescents with ASD have employed the SRS-2 as the primary efficacy outcome measure, commonly scored via parent proxy report (SRS-2) [9, 17, 24, 35]. Consequently, the value adolescents with ASD themselves place on SSGT interventions and to what extent these interventions enable them to achieve personally meaningful social goals is unclear. To address this gap, the current RCT will support adolescents to set personalised goals, evaluating the efficacy of KONTAKT© through employing GAS as the primary outcome.

Previous research evaluating the quality of life of adolescents in response to SSGT has exclusively evaluated quality of life at assessment time points, most frequently at baseline, post-test and follow up, with the daily or ‘real-time’ feelings of adolescents during and after the intervention period remaining unknown [90]. To support a better understanding of the everyday feelings of adolescents during the intervention and follow-up periods, this RCT will measure participants’ daily emotions on five dichotomised emotional states (chosen by the researchers for the purposes of this study) via self and parent proxy report using experience sampling methods. This data will enable investigation of the short-term and long-term emotional trajectories of participants. There is increasing interest in whether an intervention is capable of eliciting change in biomarkers associated with ASD, one of which is eye gaze. Previous research suggests that people with ASD focus more on the mouth than eyes during emotion recognition, negatively impacting their accuracy [91]. This study will investigate for the first time the impact of the KONTAKT© SSGT programme on adolescent’s eye-gaze behaviour when viewing facially expressed emotions and their knowledge of basic and complex emotions.

### Active control

RCTs evaluating SSGT have largely based determinations of their efficacy on comparison with either waitlist or no-treatment control groups [92, 93]. This represents a significant limitation in research design, given these designs do not support investigation of improvements in reported outcomes resulting solely from exposure to a peer group context without an explicit social skills training intervention. The design of the present study addresses this limitation controlling for exposure to a group context, by comparing KONTAKT© to an active manualised control group, Super Chef. This manualised cooking programme will engage participants in tailored shared activities in a supportive social context, without overt teaching of social skills [94].

### Cost analysis

Given that health service delivery internationally is increasingly impacted by economic rationalism, questions relating to the cost utility and effectiveness of interventions are also increasingly important to researchers, service providers, and policy makers. In the Australian context, a shift from a block funding model to the National Disability Insurance Scheme (NDIS), a model whereby disability funds are directed by people with disabilities themselves and their advocates, represents a seismic shift in the model of disability funding nationally [95]. In this context, understanding the economic costs and benefits of interventions such as KONTAKT© is relevant not only to government agencies and services providers, but also to individuals living with ASD and their families. While several interventions in ASD have shown moderate to large effect sizes [9, 28–30, 34], the absence of a cost analysis makes it difficult for all stake holders to estimate their cost-effectiveness, either in the short or longer term [96]. In addressing this limitation, the current study will obtain data on health-related costs for both the intervention and control groups. While the cost-effectiveness of KONTAKT© will be evaluated over a relatively short time (9 months), this will provide valuable information for consumers and government agencies in resource planning decisions.

### Masking and blinding

Recent reviews of the literature [19, 96] highlight a need for rigorous and adequately powered studies with improved methodological approaches to support understanding of the efficacy of SSGT interventions. One of the most consistently noted limitations of research in this field is the over reliance on proxy reports from unblinded parents, with results likely influenced by expectancy bias [96]. In addressing this issue, this study will use a blinded assessor at the three data collection time points. As suggested by the CONSORT guidelines [48], this will add to the rigour of the study. In addition, during the data collection session, the participants were instructed not to reveal to the assessor the group to which they were allocated.

### Dosage

Previous comparison between the 24-week and 12-week versions of KONTAKT© in Sweden demonstrated that the longer version was significantly more effective (ES = 0.8) than the shorter version (ES = 0.3) [17, 32] in reducing ASD symptomatology. This finding suggests that extended training results in greater social skills improvement in adolescents with ASD, pointing towards a dosage effect. As in the present protocol, the evaluation of KONTAKT© in Sweden adopted a mixed methods approach to evaluating its efficacy, which revealed that parents desired ongoing social skills support for children with ASD [97]. The current study will provide further insights into the efficacy of a “midway” dosage programme, with the number of prescribed sessions falling between the dosage provided in the short and longer versions of KONTAKT©.

### Service providers

Programme delivery in the current RCT will be administered by the AAWA at four community-based centres, which fall under the umbrella of a large community-based service provider of ASD-related services. As such this evaluation will be pragmatic, enabling understanding of the efficacy of KONTAKT© in the context of a community-based organisation. Research evaluating the efficacy of previous SSGT programmes has been criticised for its propensity to base delivery of these programmes at university clinics, which likely inadvertently biases samples towards a higher socio-demographic [19].

### Adverse effects

Even in the presence of significant group differences, some evidence suggests that psychological interventions such as SSGT may not be universally effective, with the chance that a minority of the participants may not benefit or even be harmed by the intervention [82]. This poses a significant problem and is an area of concern that is often overlooked [98]. While several studies have attempted to capture the adverse events related to a SSGT interventions [9, 17, 35], no study has investigated these effects fully. The present study will address this limitation, asking the adolescents themselves if they experienced any negative feelings or events during or after their involvement in either programme (as measured by the NEQ). These data will provide evidence of the frequency and characteristics of adverse effects enabling refinement of future SSGT programmes.

### Data analysis

Another noted limitation of previous research is the limited use of mixed-method approaches in evaluating the efficacy of SSGTs [97]. A mixed-method approach, combining both quantitative and qualitative approaches will enable an in-depth and rich understanding of the efficacy of the KONTAKT© programme [99]. This method will also enable a comprehensive understanding of the causes contributing to individual variability in treatment outcomes and will ensure the accuracy of results.

## Conclusion

This study will employ a pragmatic RCT to evaluate the efficacy of KONTAKT© in adolescents with ASD, compared to a manualised active control group, Super Chef. The ultimate efficacy of the programme will be measured in relation to participants’ achievement of personally meaningful social goals. The design of this study will provide valuable insights into the role of exposure to a social context in supporting social outcomes, the cost-effectiveness of KONTAKT© in Australia, measuring the negative effects of the intervention, and the alteration in eye-gaze behaviour contributing to the body of knowledge on SSGTs (significantly KONTAKT©).

## Supplementary information

**Additional file 1.** Social skills group training programmes for adolescents with autism spectrum disorder: a literature review. This is the literature review and tables of it discussed in the background section.

**Additional file 2.** Standard Protocol Items: Recommendations for Interventional Trials (SPIRIT). This is a recommended item to address in a clinical trial protocol.

## Data Availability

Confidentiality of data will be maintained at all times. To allow the compilation of individual data sets at all levels of follow up, participants will be allocated an identification number, with corresponding names being maintained by the researchers in a locked filing cabinet separate from data sets. All data will be stored by the researchers in a locked filing cabinet at Curtin University for a period not less than 25 years. There is no public access to the datasets generated and/or analysed during the current study, and they are only available from the corresponding author upon reasonable request.

## Abbreviations

AAWA: Autism Association of Western Australia
ADOS-2: Autism Diagnostic Observation Schedule - second edition
ANZCTR: Australian New Zealand Clinical Trials Registry
ASD: Autism spectrum disorder
CARG: Curtin Autism Research Group
CBCL: Child Behaviour Checklist
CHU9D: Child Health Utility 9D
CONSORT: Consolidated Standards of Reporting Trials
CSIE: Circumplex Scale of Interpersonal Efficacy
DSM-5: Diagnostic and Statistical Manual of Mental Disorders - version 5
DSM-IV: Diagnostic and Statistical Manual of Mental Disorders - version IV
ERSSQ: Emotion Regulation and Social Skills Questionnaire
ESM: Experience Sampling Method
ES: Effect size
GAS: Goal Attainment Scaling
ICER: Incremental Cost-Effectiveness Ratio
IQ: Intelligence Quotient
NEQ: The Negative Incidents and Effects of Psychological Treatment
PLS: Perth Aloneness Scale
PedsQL 4.0: Pediatric Quality of life Inventory - fourth edition
QALY: Quality-Adjusted Life Years
RCT: Randomized controlled trial
RED: Remote eye tracker device
SDAC: Survey of Disability, Aging and Carers
SIAS: Social Interaction Anxiety Scale
SPSS: Statistical Package for the Social Sciences
SRS-2: Social Responsiveness Scale - second edition
SSGT: Social skills group training
TIC-P: Treatment Inventory of Costs in Patients
TSS-2: Treatment Satisfaction Scale
WASI-II: Wechsler Abbreviated Scale of Intelligence - second edition
