# Supplementary material for: KONTAKT© for Australian adolescents on the autism spectrum: protocol of a randomized control trial
Source: Trials. 2019 Dec 9;20:687. doi: 10.1186/s13063-019-3721-9 (PMC6902510; doi:10.1186/s13063-019-3721-9)
Supplement: Supplementary file 1 — Additional file 1. Social skills group training programmes for adolescents with autism spectrum disorder: a literature review. This is the literature review and tables of it discussed in the background section. [file 13063_2019_3721_MOESM1_ESM.docx]

**Additional file 1: Social skills group training programs for adolescents with Autism spectrum disorder: A literature review**

This current literature review examines detailed information from the current manualized social skills group training programs for adolescents with Autism spectrum disorders which have been evaluated in a Randomized Control Trial.

| Table 1- Inclusion, exclusion, tolerated comorbidities, recruitment strategy and setting characteristics in previous SSGTs for ASD | | | | | | | | | | | | | | |
| --- | --- | --- | --- | --- | --- | --- | --- | --- | --- | --- | --- | --- | --- | --- |
| First author/ publication year/ location | Inclusion Criteria | | | | | | | | Exclusion Criteria | | Comorbidities | | No. of Centres  Recruitment strategy  Centre characteristic | |
|  | N | IQ | Age | Diagnosis | Gender  F/M | Other | | |  | |  |  |  |  |
| Corbett, B  2016  USA | 30 | IQ >70 | 8-14 | ASD | 7/24 | - | | | - | | - | | -/Y/- | |
| Frietag, C  2016  Germany | 209 | IQ >70 | 8-19 | Autistic, AS, atypical autism | 15/186 | German Fluency (Child and carer) | | | Schizophrenia, Bipolar disorder, SP, OCD, MDD with suicidal thoughts psychiatric disorders, aggressive behaviour, neurological disorders (except well treated epilepsy), medical conditions interfering with therapy, participation in other clinical trials | | ADHD, ODD, anxiety excluding social anxiety | | 6/Y/Y | |
| Gabriels, R  2015  USA | 127 | IQ>80 | 6-16 | ASD | 15/105 | SCQ>15; ABC-C>11 | | | Genetic disorder; history of medical or behavioral issues, animal abuse, phobia of horses, more than 2 hours of exposure to such therapies within the past 6 months, weight exceeding the riding center’s limits, Siblings (with ASD) of included participants | | Mood disorder, anxiety disorder, ADHD, learning disability | | -/Y/- | |
| Jonsson, U  2018  Sweden | 50 | IQ >70 | 8-17 | ASD | 15/35 | Motivated to participate | | | Clinically assessed self-injury, conduct disorder, anti-social personality disorder, borderline personality disorder and any psychotic disorder | | ADHD, anxiety, mood disorder | | 2/Y/Y | |
| Laugeson, E  2009  USA | 33 | IQ >70 | 13-17 | HFA, AS, PDD-NOS | 5/28 | English fluency, no history of major mental illness (e.g. bipolar disorder schizophrenia, or psychosis), absence of hearing, visual, or  physical impairments | | | - | | Receiving prescribed psychoactive medication | | -/Y/- | |
| Lerner, M  2012  USA | 13 | - | - | Asperger, Autism, PDD-NOS | 0/13 | - | | | - | | - | | -/-/- | |
| Matthews, N  2018  USA | 34 | IQ >70 | 13-17 | Autism, ASD | 6/28 | Spending at least 80% of educational time in general education setting, difficulty making friends according to parent report, parent willingness to be involved | | | Home schooled or online students | | ADHD, anxiety, depression, bipolar-NOS, Mitochondrial disorder | | -/Y/- | |
| Olsson, N  2017  Sweden | 296 | FSIQ >70 | 7-12 &  13-17 | autism, AS, atypical autism, PDD-NOS | 88/208 | Motivated to participate | | | Clinically assessed self-injury, conduct disorder, anti-social personality disorder, borderline personality disorder and any psychotic disorder | | ADHD, anxiety, mood disorder | | 13/Y/Y | |
| Schohl, K  2014  USA | 58 | verbal  IQ > 70 | 11-16 | ASD | 11/47 | English fluency (child and carer), no history of major mental illness (bipolar disorder, schizophrenia, or psychosis), hearing, visual, or physical impairments interfering with participation in activities | | | - | | N/A | | -/Y/- | |
| Vernon, T  2017  USA | 40 | verbal  IQ > 70 | 12-17 | ASD | 11/24 | - | | | Not having ASD diagnosis | | N/A | | -/Y/- | |
| White, S  2013  USA | 30 | verbal  IQ > 70 | 12-17 | Autism, AS, PDD-NOS | 7/23 | - | | | Primary diagnosis of OCD, PD, PD with agoraphobia, or agoraphobia without PD. Serious behaviour issues | | SoP, GAD, SP or SAD, OCD, PD/Agor, PTSD. | | 1/Y/Y | |
| Yoo, H  2014  Korea | 47 | verbal  IQ >65 | 12-18 | PDD-NOS, Autistic disorder , AS | 3/44 | | Motivated to participate, no history of major mental illness, no current aggressive behaviour or severe oppositional tendency, no hearing, visual or physical or neurological disabilities interfering with participation in activities | - | | N/A | | -/Y/- | |  |

**ADHD**: Attention Deficit Hyperactivity Disorder; **Agor**: Agoraphobia; **AS**: Asperger Syndrome; **ODD**: Oppositional Defiant Disorder; **GAD**: General Anxiety Disorder; **HFA**: High Functioning Autism; **MDD**: Major Depression Disorder; **OCD**: Obsessive-Compulsive Disorder; **PD**: Panic Disorder; **PDD-NOS**: Pervasive Developmental Disorder; **PTSD**: Post-Traumatic Stress Disorder; **SAD**: Separation Anxiety Disorder; **SoP**: Social Phobia; **SP**: Special Phobia

Table 2 – Intervention and comparison group characteristics in previous SSGTs for ASD

| Reference | Intervention group | | | | | | | | | Comparison group | | Trainer's Characteristics  /Training  /Supervision | |  |
| --- | --- | --- | --- | --- | --- | --- | --- | --- | --- | --- | --- | --- | --- | --- |
|  | Manual | | Session | | N trainers | N  per group | Fidelity measure | Individualized activity | Homework/  generalization activity | Type | N |  |  |  |
|  |  |  | N | Duration  (min.) |  |  |  |  |  |  |  |  |  |  |
| Corbett, B  2016 | SENSE | | 10 | 240 | 12 | 17 | Y | Y | Y/Y | Usual Care* | 16 | Y/Y/- | |  |
| Frietag, C  2016 | SOSTA-FRA | | 12 | 90 | 2 | 4-5 | Y | - | Y/Y | Delayed Treatment | 102 | Y/Y/Y | |  |
| Gabriels, R  2015 | Therapeutic Horseback riding | | 10 | 45 | At least 1 per participant | 2-4 | Y | - | -/- | Active control | 47 | -/-/- | |  |
| Jonsson, U  2018 | KONTAKT | | 24 | Children  :60  Adolescents: 90 | 2-3 | 4-8 | Y | Y | Y/Y | Usual Care | 27 | Y/Y/Y | |  |
| Laugeson, E  2009 | PEERS | | 12 | 90 | - | 7 | Y | Y | Y/- | Delayed Treatment | 16 | Y/Y/Y | |  |
| Lerner, M  2012 | SDARI | | 4 | 90 | - | 7 | Y | - | Y/- | Active control | 6 | -/Y/Y | |  |
| Matthews, N  2018 | Peer mediated PEER | | 14 | - | 2 | - | Y | - | Y/- | Active control + Delayed treatment | 12/13 | Y/Y/Y | |  |
| Olsson, N  2017 | KONTAKT | | 12 | Children:60  Adolescents: 90 | 2-3 | 4-8 | Y | Y | Y/Y | Usual Care | 146 | Y/Y/Y | |  |
| Schohl, K  2014 | PEERS | | 14 | 90 | 1 or 2 | < 10 | Y | N/A | Y/- | Delayed Treatment | 29 | Y/Y/Y | |  |
| Vernon, T  2017 | START | | 20 | 90 | - | 3-6 | Y | - | Y/Y | Delayed Treatment | 19 | Y/Y/Y | |  |
| White, S  2013 | MASSI | 13: individual, 7: group | | Groups: 75  Individual:  60-70 | - | 15 | Y | Y | -/- | Delayed Treatment | 15 | Y/Y/Y | |  |
| Yoo, H  2014 | PEERS | | 14 | 90 | - | 6-10 | Y | N/A | Y/- | Delayed Treatment | 24 | | Y/Y/N | |

* The Waitlist later received the intervention but no data was gathered from them

Table 3 – Timing of assessment, blinding, outcome measures, incentives, cost analysis, results and report of adverse events in previous SSGTs for ASD

| Reference | Assessment time points | Blinding (Masking) | **Primary**/secondary report outcomes | incentive | Cost analysis | Results in comparison to control group | adverse event | |
| --- | --- | --- | --- | --- | --- | --- | --- | --- |
| Corbett, B  2016 | Baseline  Post- test  (2-months follow-up)* | Coder | Parent proxy: SRS, ABAS  Research team: PIP, MFI, MFD, ToM, ERP, Incidental Face memory Task | - | - | Improvement in social cognition | - | |
| Frietag, C  2016 | Baseline  mid-intervention  post-test  3-month follow up | teachers | Adolescent: DIKJ, Therapy quality and achievements  Parent proxy: **SRS**, CBCL, SDQ  Teacher: SRS, SDQ  Research team: change in EEG activity | token program | - | Improvement in parent proxy SRS (predominantly in male HFA participants) | Y | |
| Gabriels, R  2015 | Baseline  Post-test | Assessors | Speech therapist: PPVT-4, SALT, BOT-2, SIPT  Parent proxy: ABC-C, SRS | - | - | Improvement in social cognition and communication in the treatment group | - | |
| Jonsson, U  2018 | Baseline  post-test  12 week follow-up | - | Adolescent: CiS  Parent: PSS  Parent proxy: **SRS**, ABAS-II  Teacher: SRS, ABAS-II,  Research team: DD-CGAS, CGI-S | €12 voucher | - | Larger improvement than previously  reported for shorter SSGT suggesting added benefits of extended program with optimizing the delivery of SSGT |  | |
| Laugeson, E  2009 | Baseline  Post-test | teachers | Adolescent: QPQ, TASSK-R, FQS  Parent proxy: SSRS-P, QPQ  Teacher: SSRS-T | Light meal with beverage during each session | - | Improvement in knowledge of social skills, increased frequency of hosted get-togethers, and improved overall social skills as reported by parents | - | |
| Lerner, M  2012 | Baseline  Post-test | Parent | Adolescent  Parent proxy: SCQ, SRS, SSRS-P  Research Team: SIOS  Teacher: SSRS-T | - | - | Both groups increased in reciprocated friendship nominations and staff-reported social skills | - | |
| Matthews, N  2018 | Baseline  post-test  20-week follow-up | - | Parent proxy: SRS, SSIS  Adolescent: TASSK, SIAS, R-UCLA, QSQ, AKQ | $150 for appreciation of participation | - | modest advantage in social skills knowledge and social functioning for participants in the peer-mediated  PEERS curriculum | - | |
| Olsson, N  2017 | Baseline  post-test  3-month follow-up | Teacher | Adolescent: CiS  Parent: PSS  Parent proxy: **SRS**, ABAS-II  Teacher: SRS, ABAS-II,  Research team: DD-CGAS, CGI-S | $12 voucher | Just total cost for the trial | Improvements on autism-related symptomatology and adaptive functioning in adolescents and females. Effects observed only by unblinded parents. | post treatment satisfaction questionnaire | |
| Schohl, K  2014 | Baseline  Post- test | Teacher | Adolescent: TASSK-R, QSR, FQS, SIAS  Parent proxy: QSQ, SRS, SSRS-P  Teacher: SRS, SSRS-T | $30 prize | - | Improvements in knowledge of PEERS concepts and friendship skills, increased number of get-togethers, decreased social anxiety, core autistic symptomatology and problem behaviours | - | |
| Vernon, T  2017  USA | Baseline  Post-test | - | Adolescent: SSIS, SMCS  Parent: SSIS, SRS-2, SMCS | - | - | Improvement across all measures used in the study. |  | |
| White, S  2013 | Baseline  Post-test | DDGAS by an unaffiliated assessor | Adolescent: Intervention satisfaction rating  Parent: intervention satisfaction rating  Parent proxy: **SRS**, **CASI-Anx**  Research team: Paediatric anxiety rating scale, clinical global impressions-improvement scale | - | - | More than half shown improvement on parent proxy SRS | spontaneously reported by parents | |
| Yoo, H  2014 | Baseline  Post- test  3-month follow-up | - | Adolescent: **TASSK-R**, **QPQ**, K-SSRS, **SCQ**, CDI, STAIC-T, STAIC-S  Parent: BDI, STAIC-T, STAIC-S, **ASDS**  Parent proxy: QPQ, **SRS**, ASDS, K-CBCL  Research team: **ADOS**, **EHWA-VABS** | - | - | Improvement in communication and social interaction (ADOS), interpersonal relationships and leisure time (Korean vineland), social skills knowledge total scores on TASSK-R and decreased depressive symptoms. Parents had reduced maternal state anxiety. | - | |
| Outcome measures: **ABAS-II:** Adaptive Behaviour Assessment System II; **ADOS**: Autism Diagnostic Observation Schedule; **AKQ**: Autism Knowledge Questionnaire; **ASDS**: Asperger Syndrome Diagnostic Scale; **CASI-Anx**: Child and Adolescent Symptom Inventory-4 ASD Anxiety scale; **BOT-2:** Bruininks–Oseretsky Test of Motor Proficiency–2nd Edition; **CBCL**: Child Behaviour Checklist; **CDI**: Child Depression Inventory; **CGI-S**: OSU Autism Clinical Global impression – Severity; **CHAOS**: Confusion, Hubbub, and Order Scale; **CiS**: Children in Stress; **DD-CGAS**: Developmental Disabilities Children’s Global Assessment; **DIKJ**: Depressioninventar fur Kinder- und Jugendliche; **EHWA-VABS**: Korean version of the Vineland Adaptive Behaviour Scale; **FQS**: Friendship Qualities Scale; **K-CBCL**: Korean version of Childhood Behaviour Checklist; **K-SSRS**: Korean version of Social Skills rating system; **MFI**: Memory for faces (Immediate); **MFD**: Memory for faces (Delayed); **PIP**: The Peer interaction Paradigm; **PSS**: perceived Stress Scale; **QPQ**: Quality of Play Questionnaire; **QSQ**: Quality of Socialization Questionnaire; **PPTV**: Peabody Picture Vocabulary Test, Fourth Edition; **R-UCLA**: Revised UCLA Loneliness Scale; **SALT**: Systematic Analysis of Language Transcripts; **SCQ**: Social Communication Questionnaire; **SDQ**: Strength and difficulties Questionnaire; **SIAS**: Social Interaction Anxiety Scale; **SIOS**: Social interaction observation system; **SIPA**: Stress Index for Parents of Adolescents; **SIPT**: Sensory Integration and Praxis Test; **SMCS**: Social Motivation and competencies Scale); **SRS**: Social Responsiveness Scale; **SSRS**: Social Skills Rating System; **STAIC-S**: The Trait Anxiety Inventory for Children; **STAIC-T**: The State Anxiety Inventory for Children; **TASSK-R**: Test of Adolescent Social Skills Knowledge _ Revised; **ToM**: Theory of Mind; **VABS-II**: Vineland Adaptive Behavioral Scales–2nd Edition  * Only for SRS and ABAS | | | | | | | |  |

Table 4 – Timing of assessment, blinding, outcome measures, incentives, cost analysis, results and report of adverse events in previous SSGTs for ASD

| Reference | Compliance with CONSORT Statement | Intent to treat data analysis | Randomization process | Analysis | Allocation concealment |
| --- | --- | --- | --- | --- | --- |
| Corbett, B  2016 | CONSORT required information | - | Y | ANCOVA | - |
| Frietag, C  2016 | - | - | Y | Mixed  Model Repeated Measures (MMRM) | - |
| Gabriels, R  2015 | - | Y | Y | Linear mixed-effects | - |
| Jonsson, U  2018 | CONSORT diagram | - | Y | Linear mixed-effect | - |
| Laugeson, E  2009 | - | - | - | mixed MANOVA approach | - |
| Lerner, M  2012 | - | - | - | repeated-measures ANOVA Linear modelling–growth | - |
| Matthews, N  2018 | - | - | - | MANOVA models | - |
| Olsson, N  2017 | CONSORT diagram | - | Y | Linear mixed-effect | - |
| Schohl, K  2014 | CONSORT diagram | - | - | Repeated measure MANOVA | - |
| Vernon, T  2017 | CONSORT diagram | - | - | Mixed MANOVA | - |
| White, S  2013 | CONSORT diagram | Y | - | ANCOVA | - |
| Yoo, H  2014 | - | Y | Y | Repeated measures ANOVA | - |
